# Where Do Introns Come From?

**DOI:** 10.1371/journal.pbio.0060283

**Published:** 2008-11-25

**Authors:** Francesco Catania, Michael Lynch

## Abstract

The systems for mRNA surveillance, capping, and cleavage/polyadenylation are proposed to play pivotal roles in the physical establishment and distribution of spliceosomal introns along a transcript.

In eukaryotes (and viruses), genes may be organized into coding and noncoding regions, called exons and (spliceosomal) introns, respectively (Box 1). Both types of sequences are transcribed into pre-mRNA, but whereas exons are used for protein synthesis, introns are spliced out during/immediately after transcription [1] (Figure 1). Although spliceosomal introns are widespread in the eukaryotic tree, they are unequally distributed across species as a consequence of ongoing intron gain and loss [2,3]. So for instance, 287 spliceosomal introns populate the entire genome of the baker's yeast (Saccharomyces cerevisiae) [4], but this number increases to ~4,760 in a different yeast species (Schizosaccharomyces pombe) and reaches ~38,000 and ~140,000 in the genomes of the fruit fly Drosophila melanogaster and Homo sapiens, respectively [5]. Explaining the causes and functional implications of this uneven distribution requires understanding why spliceosomal introns exist in the first place and what the evolutionary origin(s) of these sequences are—a problem that has proved a conundrum for the past 30 years [6].

**Figure 1:**
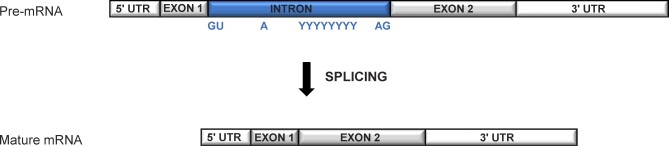
Schematic Example of an Intron-Containing Gene The regions preceding and following the coding regions (exons) are transcribed but not translated, and are called 5′ and 3′ UTRs. The intronic sequence intervenes between the two coding exons and contains splicing signals that are recognized by a nuclear machinery, the spliceosome, which carries out splicing. Splicing signals are located at both ends of the intron (e.g., canonical GU and AG dinucleotides at the 5′ and 3′ splice site, respectively) and within the intron (an adenine residue, called the branch site, and frequently a polypyrimidine (C/U) tract.

**Box 1. Spliceosomal Introns**

Spliceosomal introns are noncoding intervening sequences of eukaryotic and viral genes that are removed during the process of pre-mRNA maturation, leaving only coding sequence (exons) to be part of the messenger RNA. Three additional classes of introns are known—the group-I, group-II, and group-III introns, all of which are capable of self-splicing. Spliceosomal introns cannot self-splice but are removed by a dynamic nuclear apparatus, the spliceosome. Such machinery typically consists of five uridylate-rich small nuclear RNAs (U1, U2, U4, U5, and U6) and a large number of associated proteins.

## Did Spliceosomal Introns Insert in or Emerge from Coding Sequences?

Although the evidence is circumstantial, it is widely thought that spliceosomal introns originated from group-II introns—self-splicing introns that are widely found in fungi, plants, protists, and bacteria—which invaded the uninterrupted nuclear genes of an early eukaryote and subsequently lost the ability to self-splice, as the host genome took over this function [7–10]. However, while group-II introns may have spawned the primordial population of spliced introns and the present-day mechanism for their removal (the spliceosome), this model does not provide an explanation for either the irregular distribution of spliceosomal introns in modern-day species [11] or for recent episodes of intron gain [12–14].

Recent studies on vertebrate genome evolution have shown that numerous exons have emerged from pre-existing noncoding sequences (i.e., introns) [15,16]. The acknowledged de novo generation of exons from introns—termed “exonization”—leads us to ask the question: Does the inverse process, i.e., “intronization” or the creation of spliceable sequences from nonintronic regions, take place? A number of empirical observations suggest that intronization is more than a formal possibility [17–23] (see below).

## When Nonsense Codons Become Meaningful

The process of translation normally ends when the ribosome reaches a nonsense (or stop) codon at the end of the coding mRNA. A nonsense codon that is positioned upstream of the true stop can lead to premature translation termination and thus is called a premature termination codon (PTC). PTCs found in eukaryotic coding sequences are sometimes excluded from the mature mRNA [24–31], for example by the activation of otherwise latent 5′ splice sites that act to remove the PTC from the transcript [32]. In addition, many eukaryotic PTC-containing mRNAs that are not subject to these nuclear mechanisms of PTC recognition and exclusion are nonetheless subject to degradation in the cytoplasm by the nonsense-mediated decay (NMD) pathway [33]. Notably, as PTC-harboring alleles may have harmful phenotypic effects—since aberrant gene products can lead to cell damage—these mechanisms provide potential cellular routes for reducing the negative effects of PTC-containing alleles. While secondary mutations that re-establish the reading frame are possible, changes that increase the efficiency of spliceosomal removal of the PTC will—at a minimum—also return the mRNA dosage to normal.

## The First Steps of the Intronization Hypothesis

We suggest that the cell's capacity to filter out aberrant transcripts, in concert with imperfect splice-site recognition [34,35], may provide a powerful mechanism for generating spliceosomal introns. Specifically, if a PTC-containing exonic region is accidentally spliced out during mRNA maturation, and the open reading frame (ORF) of the transcript is preserved, the NMD pathway will not be elicited by that transcript, providing the key first step in the establishment of a new intron.

In this model, we propose that protein-coding sequences that fortuitously contain the minimal requisite sequence information for spliceosome-mediated recognition (see splicing signals in Figure 1) have a latent potential to undergo splicing and to intronize after acquiring PTC mutations that interrupt the ORF of the message. Although splicing to remove a PTC may initially be inefficient, degradation of the pool of unspliced PTC-containing transcripts by NMD will produce a relatively pure pool of PTC-free mature mRNAs [21], thereby maintaining the spliced allele in a possibly still active state. Such an allele can then be subject to positive selection for subsequent mutations that improve splicing of the modified region.

Mutant alleles with PTC-compensating splicing are more likely to become established if they generate proteins that retain at least some activity. Thus, we expect introns arising from this process to share certain characteristics: (1) a short length, thereby minimizing the number of lost codons; and (2) a sequence length that is a multiple of three, so as to preserve the ORF. Also, as the emergence of introns in small exons would lead to the creation of two even smaller flanking exons, whose correct splicing might be compromised [36], we expect either that introns never (or rarely) emerge in short exons or that intronization includes the whole exon. In the latter case, notably, if the small exon is not terminal, the intronization process would lead to the merger of two introns and the encompassed exon and hence to the loss, rather than a gain, of an intron. Unless excision of the newly intronized coding sequence has sufficiently large deleterious consequences, the fixation of the novel intron may be either selectively neutral or promoted by natural selection.

## The Role of Alternative Splicing in the Process of Intronization

As it is unlikely that the process of intronization is instantaneous, we predict the existence of a transient phase during which gene regions are neither fully exonic nor fully intronic, essentially exhibiting the features of DNA sequences undergoing alternative splicing. Alternative splicing is a nuclear process that leads to the incorporation of noncoding regions and/or the exclusion of coding regions from mature mRNAs [37], events that bear on the original definition of exon and intron [6].

**Figure 2:**
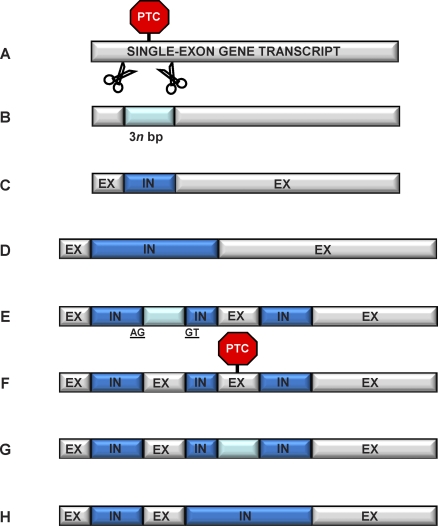
Schematic Example for a Hypothesized Temporal Succession of Exonization and Intronization Processes (A) A PTC-containing exonic region is fortuitously spliced. (B) If the region spliced has a length that is a multiple of 3, and its absence is not detrimental to the functionality of the coded protein, the exonic region may start being skipped, with its incorporation in the mature mRNA gradually decreasing, essentially undergoing a phase of alternative splicing (indicated in light blue). (C) The constitutive intron (IN) is created, flanked by two constitutive exons (EX); and (D) may grow larger (for example, as a consequence of insertion accumulation). (E) A second constitutive intron arises downstream in a way similar to that described for the previous intron, while a process of exonization starts within the previous intron. (F) The novel, initially alternatively spliced exon now becomes constitutively spliced, while the exon downstream of it acquires a PTC and may start the process of intronization. (G) The intronization process of the PTC-containing exon undergoes a phase of alternative splicing, which subsequently leads to (H) the technical loss of one intron and one exon and the creation of a single, larger intron.

Several findings support the hypothesized gradual conversion from exonic to intronic sequences. Specifically, across vertebrates, constitutively spliced introns in one species often align to homologous alternatively spliced coding sequences of orthologous genes from another species [16]—suggesting a source–product relationship between the two types of sequences; and alternatively spliced exons in vertebrates have been shown to emerge from constitutive exons [38,39]. Similarly, alternatively spliced introns have been found to share multiple features with exonic sequences in humans [40] and to emerge from constitutive exonic sequences in nematodes [41]. All these observations are consistent with an evolutionary loop and a shared evolutionary history between at least a subset of exons and introns (Figure 2).

The gradual conversion that we envision for the process of intronization has been reported also for the process of exonization [42]. In particular, most exons that have emerged from noncoding sequences are alternatively spliced, often being minor forms, i.e., exons that are rarely included in the mature transcript [15,16,43–47]. It is interesting to note that DNA regions undergoing exonization are commonly identified under the assumption of no parallel exon losses across species (e.g., [16]). The validity of this assumption is uncertain, and if parallel events of exon loss are relatively frequent, then several reported cases of exon gain would have to be recategorized as exon losses, i.e., events of intronization of coding sequences. The latter scenario is consistent with some remarkable feature similarities of putatively young (minor-form) exons and young introns, as predicted by the intronization model. Specifically, minor-form exons tend to be: 3*n* in size when located within the coding region [48]; unusually short; fast-evolving; PTC-enriched [49,50]; and located in polypeptide segments that have no or very little immediate effect on protein structure [51,52]; they also have weaker splice sites compared to constitutively spliced exons [53–60].

## Introns as a Result of the Crosstalk between mRNA-Associated Processes

The extensive network of interactions between mRNA-associated processes [61] suggests that other mechanisms, in addition to NMD, may be involved in the origin (and evolution) of spliceosomal introns. Here, we examine the role played by cleavage/polyadenylation factors (CPFs) and the mRNA capping-binding complex (CBC). CPFs bind 3′ untranslated region (UTR) sequence signals (positioned just past the stop codon in the coding region) and are actively involved in mRNA 3′ end formation, a process that broadly consists of cleaving the nascent transcript and adding a tail of multiple adenines to its 3′ end. The CBC is a structure that is added to the mRNA 5′ end immediately after the start of transcription and regulates several steps of mRNA metabolism [62].

Several connections have been found between the processes of cleavage/polyadenylation and splicing [63–71], and splicing factors (SFs) and CPFs have also been documented to compete or interfere with each other [72–75]. Although the targets of such competition remain unknown, in plants AU-richness, and U-richness in particular, appears to be not only a landmark for intron recognition but also a signal for CPFs [19,76–78]. The latter finding is consistent with U-rich sequences directing transcription termination in several eukaryotes and viruses [79]. Notably, U-rich sequences, such as the polypyrimidine tract (Figure 1), are also present in most eukaryotic introns and play a significant role in the splicing process [80].

**Figure 3:**
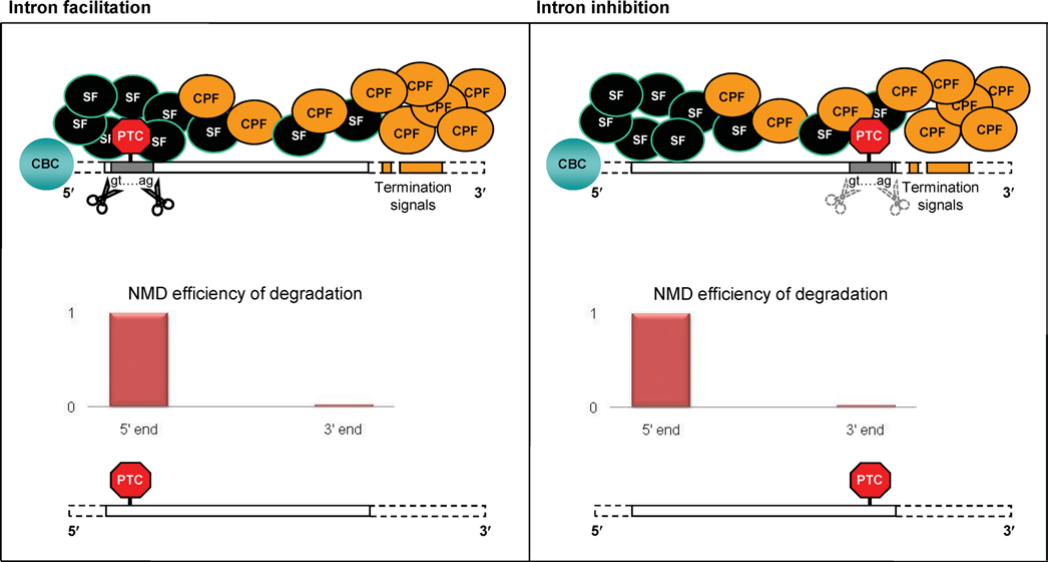
A PTC in a Coding Region Typically Elicits a Translation-Dependent Surveillance Mechanism, Such As NMD, Which Leads to the Degradation of the Aberrant Transcript If the mRNA region containing the PTC harbors fortuitous recognition elements for its spliceosome-mediated removal (e.g., latent splice sites), a PTC-containing segment may be spliced out during mRNA maturation (in grey). The likelihood with which accidental splicing of an entirely new intron may occur is expected to be higher in regions of the transcript where the concentration of SFs is naturally elevated (e.g., at the 5′ end, in proximity of the CBC), compared to the mRNA 3′ end, where strong canonical termination signals (in orange) favor the preferential binding of CPFs that, under the proposed model, compete/interfere with SFs for the binding of U-rich tracts. The fortuitous gain of introns is favored at the 5′ end because unspliced PTC-containing transcripts in this region are more efficiently degraded, thereby alleviating the negative cellular consequences of the PTC.

We propose that CPFs regularly access U-rich tracts along the mRNA during transcription, but are antagonized (or interfered with) by SFs when the U-rich regions are located within an intron. Notably, these two sets of factors are also known to antagonize/interfere in exons, as the U1 small nuclear ribonucleoprotein (an SF) inhibits 3′-end processing when bound to the 3′-end of a pre-mRNA in the vicinity of the cleavage-polyadenylation site [81–86]. Under our hypothesis, the interaction between SFs and CPFs in exon sequences modulates the likelihood that PTC mutations will be removed by a splicing event, thereby defining the physical setting for the facilitation or inhibition of intron colonization (Figure 3). The ability of CPFs to contact U-rich sequences and block SFs is expected to be affected by diverse factors, including the distance and the strength of the 5′ splice site [19], the presence of splicing-modulating sequences (such as splicing enhancers), the local concentration of splicing proteins [87], the transcription elongation rate [88,89], and the mRNA secondary structure [90]. Optimal splicing conditions also promote transcription elongation [91,92] and termination [65,93], whereas weaker splicing conditions facilitate the binding of CPFs, inhibiting the binding of SFs to the polypyrimidine tract.

The CBC also influences splicing, acting as a splicing enhancer by increasing the population of SFs local to the 5′ end [94–96], thus favoring splicing at this end of the transcript. At the 3′ end, the presence of strong canonical termination signals favors the recruitment of CPFs to terminal U-rich DNA stretches, thus inhibiting the potential assembly of SFs in this region. As a result, the CBC and the resultant excess of SFs are expected to enhance the frequency of fortuitous splicing events at the 5′ end, while the presence of strong termination signals is expected to reduce the frequency of fortuitous splicing events at the 3′ end.

Finally, the antagonistic interactions between SFs and CPFs are likely mediated by two other major classes of competing proteins [97], namely the serine/arginine-rich proteins and the heterogeneous nuclear ribonucleoproteins [87]. Notably, heterogeneous nuclear ribonucleoproteins have also been suggested to both participate in transcription termination and bind the polypyrimidine tract [79,98–100].

Central to our hypothesis is the observation that the more favorable splicing at the 5′ end of a gene parallels the spatial pattern of the efficiency of NMD degradation of aberrant transcripts. Specifically, NMD effectiveness is maximal when a PTC is proximal to the 5′ end of the mRNA and minimal when it resides in the most 3′ exon, close to the 3′ end of the transcript [101–104] (Figure 3). Thus, not only are splicing-eliciting PTCs expected to arise more frequently in the 5′ ends of genes, but such modifications also have the greatest chance of emerging as novel introns with minimal fitness effects.

## Support for the Intronization Hypothesis

The distribution of introns over the length of the coding sequence is consistent with the idea that NMD, as well as the interactions between CPFs and SFs, cooperatively guides the successful colonization by introns. In particular, the NMD pathway appears to have been lost in eukaryotic lineages that have no or nearly no introns [105]. While it is not possible to rule out that NMD could be simply lost in situations where introns are rare (e.g., as a consequence of genome reduction), we suggest that, as introns are not essential to the functioning of NMD [101,103,106–111], by increasing the costs of imperfect splicing, the loss of NMD produces an environment that inhibits intron colonization.

**Figure 4:**
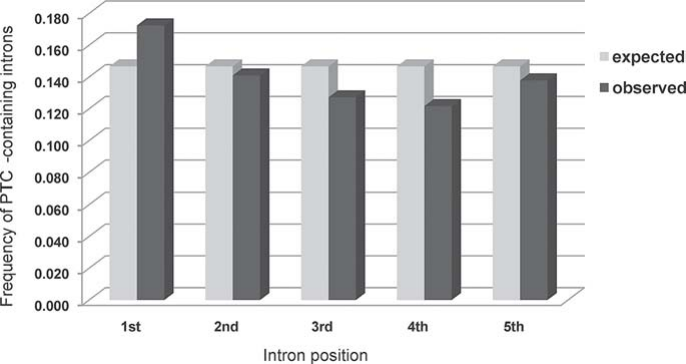
Frequency of PTC-Containing Introns in P. tetraurelia This analysis has been performed using 15,286 EST-confirmed introns [112] and does not include single-intron genes.

As for preferential intron location, assuming a steady-state process of intron birth and death, an increase in intron birth is expected to shift the age distribution to younger introns. Under our hypothesis, young introns are expected to be biased toward lengths that are multiples of three, to be relatively short, and to contain a PTC. These expectations fit the observations of a recent study where PTC-containing 3*n* introns in the ciliate Paramecium tetraurelia were revealed to be about twice as frequent compared to PTC-containing introns of the two other size classes [112]. Our own study of the intron dataset used in the latter study shows that this higher frequency is independent of the position occupied along the transcript (data not shown), and that PTC-containing introns are over-represented at the 5′ end of transcripts (1st intron position, χ^2^ = 23.26, *p* = 1.41 × 10^−6^) but less frequent toward the 3′ end (3rd intron position, χ^2^ = 7.93, *p* = 4.87 × 10^−3^; 4th intron position, χ^2^ = 6.44, *p* = 0.0111), consistent with the idea that splicing-eliciting PTCs arise more frequently in the 5′ ends of genes (Figure 4).

Our hypothesis specifically predicts that small 3*n* introns should be enriched with PTCs, either as a consequence of these stop codons eliciting intronization and/or because PTCs are secondarily selected for as a means to detect erroneously spliced transcripts. This prediction is supported by the significant under-representation of PTC-free 3*n* introns associated with short intron size in six different eukaryotes [112].

## Explaining Introns in Untranslated DNA Sequences

Within a gene, spliceosomal introns can also reside in UTRs [113], and UTR introns show similar patterns of frequency and spatial distributions in distantly related species [114,115]: 5′-UTR introns are frequent and dispersed at random, while 3′-UTR introns are very rare, despite the fact that 3′ UTRs are typically about two to three times longer than 5′ UTRs.

In light of the intronization model, these features of introns in UTRs can be explained in two non-mutually exclusive ways. First, a significant fraction of today's intron-containing UTRs may have been coding sequences at the time of intron addition. In support of this scenario, the translatability of a number of ORFs residing in currently annotated UTRs has been shown [116–119]. Second, the emergence of introns in 5′ UTRs may be associated with the potentially deleterious effects of upstream premature translation start AUG codons. Simply put, we suggest that whereas PTCs may encourage the gain of internal introns, premature translation start codons may encourage the gain of 5′-UTR external introns. The latter scenario is consistent with an elevated abundance of AUGs in 5′-UTR introns [120].

Spliceosomal introns primarily inhabit protein-coding genes, but they also sometimes interrupt noncoding RNA genes [121,122]. Although the proposed hypothesis does not claim to explain the origin of all introns, it is worth noting that the presence of spliceosomal introns in noncoding RNA genes might also be the result of accidental splicing events and of the subsequent proofreading activity of surveillance mechanisms. In particular, although no translation has been reported for the products of these genes, experimental evidence suggests that, like mRNAs, noncoding RNAs are also subject to post-transcriptional surveillance pathways [123]. A possible beneficial effect of a splicing event is the improvement in the folding of the mature RNA, and consistent with this possibility, the noncoding RNA quality-control step appears to target molecules that are either misfolded or contain functionally deleterious mutations [124–126]. Thus, as in the case of protein-coding genes, it can be postulated that fortuitous endogenous events may on rare occasions promote splicing in noncoding RNAs, in such a way as to prevent more harmful secondary structures.

## Why Don't All Eukaryotic and Viral Genes Contain Introns?

Two possible explanations for the existence of intronless genes are: (1) that introns can simply be lost, so that a subset of intron-free genes is to be expected; and (2) that some intronless genes may be derived retrogenes, i.e., mature mRNAs that are reverse transcribed into DNA copies and inserted into the genome [127]. However, splicing is known to affect mRNA export into the cytoplasm, as unspliced transcripts usually accumulate in the nucleus [128,129]. How then can transcripts of intronless genes accumulate in the cytoplasm? A number of eukaryotic and viral single-exon genes have been found to contain sequence elements that favor nucleus-cytoplasm export [130–132]. Notably, results both from in vivo and in vitro experiments show that such elements not only play a major role in nuclear export but also enhance polyadenylation and strongly inhibit splicing, thereby inhibiting intron colonization [133–135]. These findings suggest that (ancestrally or derived) intronless genes that contain the aforementioned sequence elements are unlikely to gain introns, simply because of their intrinsic resistance to the splicing apparatus. Although it remains to be proven, it is possible that the relative abundance of these elements that inhibit splicing plays a role in establishing different levels of intron-richness between eukaryotic species (e.g., between Sa. cerevisiae and Sc. pombe).

## Conclusions

We have proposed a novel hypothesis for the origin of spliceosomal introns, invoking endogenous production within translatable sequences (at least in the case of protein-coding genes), facilitated by the activity of cellular surveillance mechanisms. Despite the mutational hazard associated with intron presence and proliferation [136], we argue that, at least initially, introns might represent a favorable life line for an allele that has acquired an ORF-disrupting mutation. In this sense, in-frame stop codons need not be dead ends, as often believed, but rather sequences that occasionally facilitate the evolution of eukaryotic gene structure, possibly favoring not only intronization, but also processes such as exonization (following a PTC loss [137]). Further experimental validation of our hypothesis would not only support the idea that intron birth/death rates depend on both the population-genetic [136] and the intracellular environment, but also shed light on a surprising aspect of the evolution of eukaryotic gene structure, i.e., the ongoing, stochastic process of mutual conversion between exons and introns within genes.

## Abbreviations

CBC: capping-binding complex
CPF: cleavage/polyadenylation factor
NMD: nonsense-mediated decay
ORF: open reading frame
PTC: premature translation termination codon
SF: splicing factor
UTR: untranslated region
